# A Checkpoint-Related Function of the MCM Replicative Helicase Is Required to Avert Accumulation of RNA:DNA Hybrids during S-phase and Ensuing DSBs during G2/M

**DOI:** 10.1371/journal.pgen.1006277

**Published:** 2016-08-24

**Authors:** Sriram Vijayraghavan, Feng-Ling Tsai, Anthony Schwacha

**Affiliations:** Department of Biological Sciences, University of Pittsburgh, Pittsburgh, Pennsylvania, United States of America; Duke University, UNITED STATES

## Abstract

The Mcm2-7 complex is the catalytic core of the eukaryotic replicative helicase. Here, we identify a new role for this complex in maintaining genome integrity. Using both genetic and cytological approaches, we find that a specific *mcm* allele (*mcm2DENQ*) causes elevated genome instability that correlates with the appearance of numerous DNA-damage associated foci of γH2AX and Rad52. We further find that the triggering events for this genome instability are elevated levels of RNA:DNA hybrids and an altered DNA topological state, as over-expression of either RNaseH (an enzyme specific for degradation of RNA in RNA:DNA hybrids) or Topoisomerase 1 (an enzyme that relieves DNA supercoiling) can suppress the *mcm2DENQ* DNA-damage phenotype. Moreover, the observed DNA damage has several additional unusual properties, in that DNA damage foci appear only after S-phase, in G2/M, and are dependent upon progression into metaphase. In addition, we show that the resultant DNA damage is not due to spontaneous S-phase fork collapse. In total, these unusual *mcm2DENQ* phenotypes are markedly similar to those of a special previously-studied allele of the checkpoint sensor kinase ATR/*MEC1*, suggesting a possible regulatory interplay between Mcm2-7 and ATR during unchallenged growth. As RNA:DNA hybrids primarily result from transcription perturbations, we suggest that surveillance-mediated modulation of the Mcm2-7 activity plays an important role in preventing catastrophic conflicts between replication forks and transcription complexes. Possible relationships among these effects and the recently discovered role of Mcm2-7 in the DNA replication checkpoint induced by HU treatment are discussed.

## Author Summary

The precise regulation of DNA replication is necessary to avoid genome instability, the deleterious alteration of genetic information that is a hallmark of diseases like cancer. However, replication is more than just a simple duplication of the genetic code. The replication machinery has an additional problem–it must somehow regulate elongation to successfully elude potentially destructive conflicts with obstacles or other ongoing chromatin-dependent processes (*e*.*g*., transcription). Such conflicts generate genome instability, and how they are normally avoided is poorly understood. Using budding yeast as our model system, we show that such damage-avoidance likely requires direct involvement of the Mcm2-7 replicative helicase, the molecular motor that unwinds DNA during replication. Alteration of a key active site within the Mcm complex results in substantial levels of genome instability. Surprisingly, our evidence indicates that problems normally associated with transcription (*e*.*g*., RNA:DNA hybrids) are the ultimate source of this damage. This result implies a novel and active role for the replicative helicase in regulating elongation to successfully navigate select genomic barriers, and lays the groundwork to further explore the molecular causes and consequences of altering DNA replication in the context of genome stability.

## Introduction

Genomic instability, resulting from the loss or rearrangement of the genetic material, strongly correlates with the development of a large variety of human diseases (reviewed in [1–5]). A major source of such instability is DNA double-strand breaks (DSBs). These are thought to predominantly occur during replication through stochastic fork collapse [6–8], a process believed to result from the dissolution or inappropriate repair of stalled replication forks that have been crippled by the loss of core replication factors. Such breaks have a variety of defining features. First, they form in S-phase [8]. Second, their frequencies are aggravated by conditions that increase fork stalling (*e*.*g*., “replication stress”), a situation experimentally induced by the ribonucleotide reductase inhibitor hydroxyurea (HU) [9, 10]. Finally, specific mutations in replication fork components (*e*.*g*., loss of Mrc1, [8] and references therein) generate structurally unstable forks that coordinately increase the levels of both stochastic fork collapse and DSB formation.

Avoidance of such stochastic fork collapse during exogenous replication stress requires the DNA Replication Checkpoint (DRC). This signal transduction cascade (reviewed in [11]) consists of the Mec1/Ddc2 sensor kinase (ATR/ATRIP in metazoans), transducers (Mrc1, Tof1, and Csm3) and the Rad53 effector kinase (CHK2 in metazoans) ([11–13] and references therein). During replication stress, the DRC becomes activated, resulting in the phosphorylation of numerous downstream targets that in total protect genome integrity and reversibly block cell cycle progression until the initiating problem is repaired (*e*.*g*., [14]).

In contrast to its general inhibitory role on replication and cell cycle progression during stress, some components of the DRC may function positively to actively promote DNA replication during unchallenged growth. For example, Mec1/ATR is necessary for efficient fork progression, as *mec1/ATR* mutants in budding yeast demonstrate increased fork pausing and accumulate DSBs even in the absence of exogenous replication stress; interestingly these breaks are distinct from those resulting from stochastic fork collapse and instead share specific properties with human DNA fragile site breaks [15]. Such breaks may result from an inability to bypass specific genomic obstacles, as both Mec1/ATR and Rad53/CHK2 [16, 17] are needed to transiently uncouple physical connections between actively transcribed genes and the nuclear pore complex (‘gene gating’ (reviewed in [18])). These physical connections both directly obstruct fork progression, as well as cause an additional topological blockage by preventing free rotation of the intervening DNA ([19–21] and the references therein). In the absence of DRC function, such gated genes abnormally accumulate RNA:DNA hybrids [22] whose formation and further processing may fuel genome instability (reviewed in [23, 24]). These studies suggest that during unchallenged growth, some member(s) of the DRC is (are) required to safely modulate fork progression through specific obstacles, however the functional connection between the DRC and the core replication machinery is poorly understood.

The Mcm2-7 complex is ideally poised to coordinate DRC regulation with fork progression. Although well known as the catalytic motor of the eukaryotic replicative helicase, we have recently shown that it is also part of the DRC cascade [25]. The unusual architecture of this complex may facilitate its dual functionality. Mcm2-7 consists of six essential subunits (numbered 2 to 7) arranged in a toroidal complex, with the resulting dimer interfaces forming six distinct ATPase active sites. Biochemical evidence indicates that these six active sites contribute unequally to replication; some sites appear dedicated to DNA unwinding, while others have a likely regulatory role (reviewed in [26]). Our lab has demonstrated that a mutation which surgically ablates the function of one specific “regulatory” active site (*mcm2DENQ*) generates a separation-of-function allele: in the presence of replication stress, it blocks signal transduction of the DRC cascade, yet demonstrates essentially normal bulk DNA replication [25].

In this study, we examine the phenotypes of the *mcm2DENQ* mutant during unchallenged growth and provide evidence suggesting that Mcm2-7 is actively required to mediate conflicts between DNA replication and ongoing transcription. Using a cytological approach, we demonstrate that the *mcm2DENQ* mutant coordinately acquires high levels of DNA damage during the G2 phase of the cell cycle. By multiple criteria, we show that the observed damage is not due to stochastic fork collapse. Remarkably, the basis of this defect lies in the accumulation of RNA:DNA hybrids and the associated abnormal DNA topology, as treatments that decrease either RNA:DNA levels (over-expression of RNaseH) or abnormal DNA topology (over-expression of topoisomerase1) significantly suppress all DNA damage phenotypes. Together, our data strongly argue for a specific regulatory role of Mcm2-7 in navigating the replication fork through chromosomal obstacles. As the *mcm2DEN*Q mutation affects the apparent physical conformation of Mcm2-7 (*i*.*e*., of the Mcm2/5 gate [27]), this structural change may be an essential facet of this novel form of regulation.

## Results

### Preliminary considerations

Our entry point for this study was the checkpoint-deficient *mcm2DENQ* allele (Introduction, [25, 28]). This mutation is a substitution of the two universally conserved acidic residues of the Walker B ATPase motif in Mcm2 by their amide counterparts (Asp_606_-Glu_607_ → Asn-Gln), an alteration that abolishes ATP hydrolysis at the Mcm6/2 active site [28]. Nevertheless, in the context of the Mcm2-7 holocomplex, these changes have little to no effect on *in vitro* DNA unwinding [27]. Moreover, *in vivo*, these changes have minimal effect on Mcm2 protein expression and stability, Mcm2-7 G1 origin association, or the ability of Mcm2 to physically interact with other Mcm subunits or the DRC mediator factors Mrc1, Tof1, or Csm3 [25]. However, this mutation confers a marked defect in the DRC signal transduction cascade pathway immediately upstream of Rad53 activation [25]. To query possible replication fork collapse in the *mcm2DENQ* mutant, we analyzed the *mrc1Δ* mutant in parallel for comparison. Similar to the *mcm2DENQ* mutant, *mrc1Δ* exhibits a block to the DRC cascade immediately upstream of Rad53 activation [29]. However, in addition, the *mrc1Δ* allele also confers replication defects that lead to spontaneous fork collapse [8].

### The *mcm2DENQ* mutant exhibits genome instability and cell death during unchallenged growth

**Fig 1:**
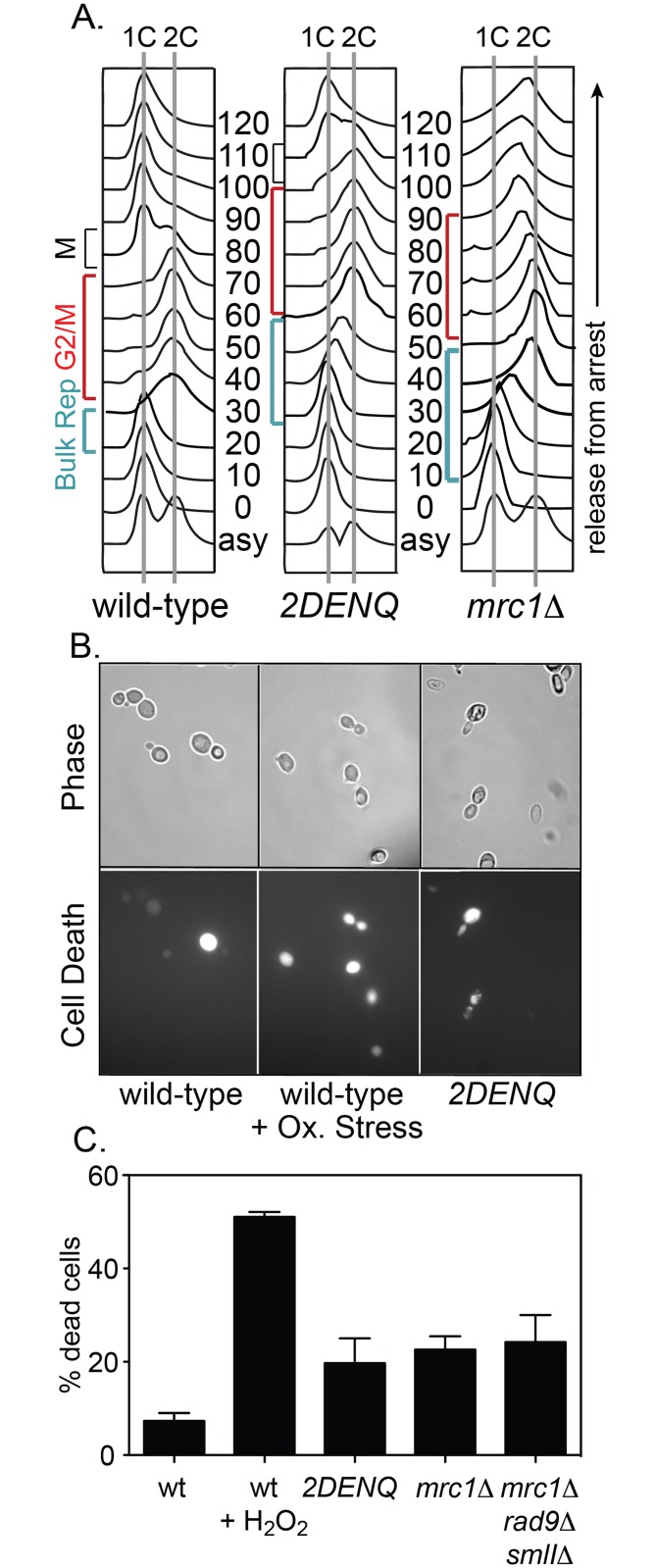
The *mcm2DENQ* mutant exhibits multiple *in vivo* defects. A) FACS analysis of wild-type (UPY464), *mcm2DENQ* (UPY499), and *mrc1Δ* (UPY713). Briefly, strains were arrested in G1 by addition of α-factor and released into fresh YPD (T = 0). Aliquots were taken at the indicated times and processed for FACS as described in Materials and Methods. B) Cell death assay. Asynchronous cultures of indicated strains from A) ± 3 mM hydrogen peroxide (positive control) were assayed for cell death (Materials and Methods). Fluorescence (i.e., cell death) and phase contrast images are shown. C) Percent of dead cells observed in the indicated strains during asynchronous growth. Strains assayed as indicated in A) plus, *mrc1Δ rad9Δ sml1Δ* (UPY715).

Unlike many *mcm* alleles of the conserved ATPase motifs that cause lethality (*e*.*g*., [28]), the *mcm2DENQ* allele broadly supports cell viability. Nevertheless, this mutant exhibits significant defects even in the absence of experimentally-induced stress (*e*.*g*., HU). As observed previously, FACS analysis demonstrates that the *mcm2DENQ* mutant grows slowly, with prolongation of both S-phase (~20 minutes) and G2/M (~10 minutes) relative to the wild-type strain (Fig 1A) [25]. Additionally, the mutant displays a modest sister chromatid cohesion defect [25].

Our current investigation now identifies two additional global *mcm2DENQ* mutant defects. First, this mutant exhibits a roughly 2.5-fold increase in the incidence of “apoptotic” cell death relative to wild-type cells, which is similar to other DRC mutants (Fig 1B and 1C) [30]. Second, the *mcm2DENQ* mutant exhibits a significantly elevated level of gross chromosome rearrangement (GCR) relative to the wild-type strain as defined by a previously-developed genetic assay [31]. This assay examines the simultaneous loss of two closely spaced genetic markers located near the non-essential left end of chromosome V (GCR events). Concomitant loss of both markers has been previously shown to be highly correlated with the occurrence of chromosome rearrangements including terminal or interstitial deletions, translocations, or chromosome fusion events, all of which are stimulated by various genotoxic chemicals or mutant backgrounds [31]. The rate of GCR events in the *mcm2DENQ* mutant is 96-fold higher than the normally low level observed in wild-type (Table 1, [32]).

**Table 1:** Analysis of Gross Chromosome Rearrangement.

Relevant genotype	strain	GCR rate/gen (# independent cultures)	p-value relative to wild-type	fold-increase
Wild-type	UPY622	2.4x10^-10^ (5)	–	1x
*mcm2DENQ*	UPY687	230x10^-10^ (3)	0.0024	96x
*mrc1Δ*	UPY698	21x10^-10^ (3)	0.0024	8.5x
*rad50Δ*	UPY694	1600x10^-10^ (6)	0.0119	667x

For comparison, parallel analysis of several additional reference alleles shows that the level of GCRs in the *mcm2DENQ* mutant is substantial: it is approximately 10-fold higher than that of a typical DRC mutant (*e*.*g*., *mrc1Δ*, Table 1, [33]), but about 7-fold less than that observed for a DSB repair mutant (*e*.*g*., *rad50Δ*, Table 1, [32]).

### The *mcm2DENQ* mutant exhibits DNA damage that peaks during G2/M

One potential cause for the elevated levels of apoptotic cells and GCRs in the *mcm2DENQ* mutant could be spontaneous DNA damage. In accord with this possibility, we have previously shown that asynchronous, unchallenged *mcm2DENQ* mutant cultures exhibit elevated phosphorylation of the DNA Damage Checkpoint (DDC) mediator protein Rad9 [25]. We confirm below using two parallel approaches that the *mcm2DENQ* mutant exhibits high levels of spontaneous DNA damage, that likely corresponds to DSBs. Moreover, we find that, despite delayed progression through S-phase, this damage appears after completion of bulk DNA replication, at G2/M.

#### -γH2AX foci

**Fig 2:**
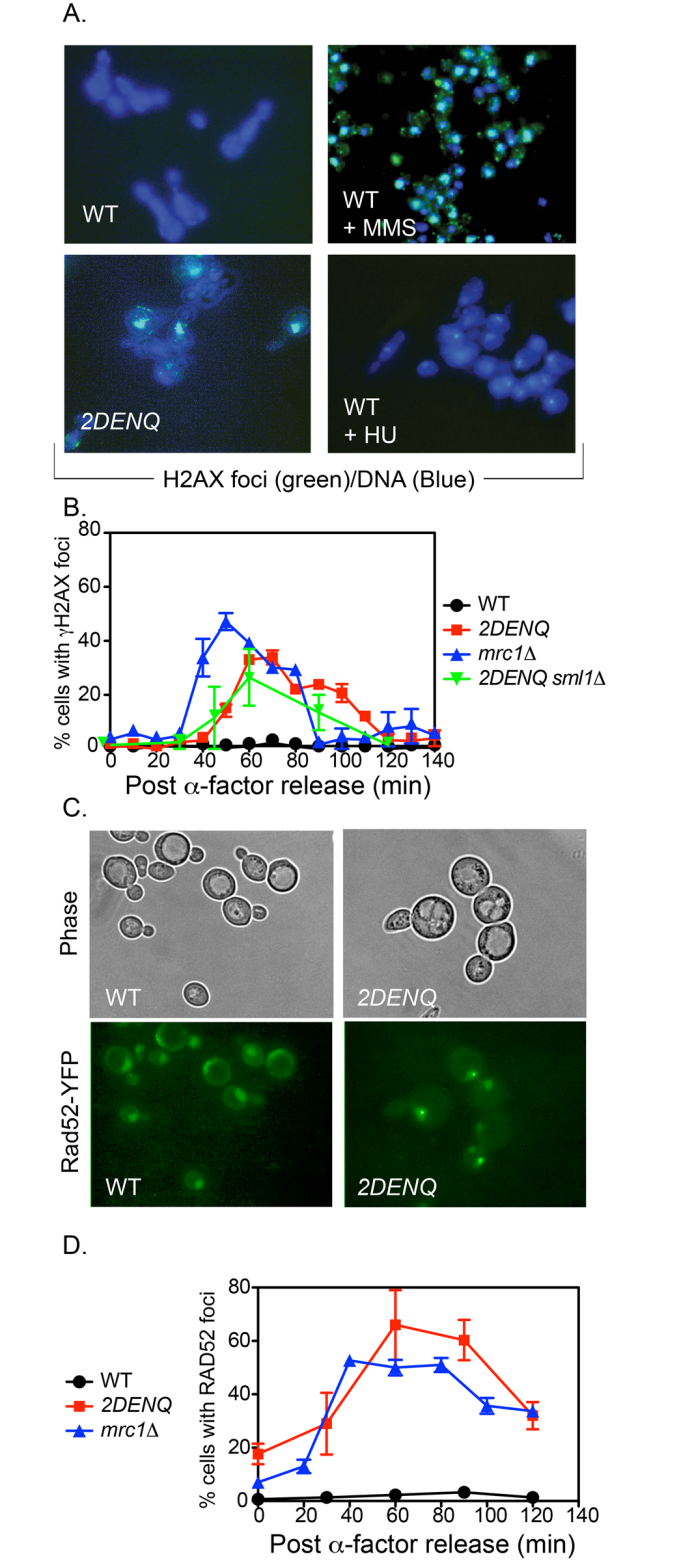
The *mcm2DENQ* allele causes DNA damage. A) γH2AX assay. Asynchronous cultures of either wild-type (UPY464) or *mcm2DENQ* (UPY499) were treated ± 0.01% MMS (to induce DSBs) or 200 mM HU (to induce replication stress) in rich media for two hours, then processed for both DAPI (blue) and γH2AX immunofluorescence (green). B) Time-course analysis of γH2AX foci during the cell cycle. Culture of strains from A), plus *mrc1Δ* (UPY713) and *mcm2DENQ sml1Δ* (UPY948) were synchronized in G1 with α-factor, released into fresh YPD, and samples were processed for γH2AX immunofluorescence. C) Rad52-YFP assay. Asynchronous cultures of either wild-type (UPY938) or *mcm2DENQ* (UPY1014) strains were processed for phase contrast and Rad52-YFP fluorescence (green) as shown. To help visualize cells in the negative control panel, fluorescence in the wild-type panel has been enhanced and should not be confused with a genuine DNA-damage signal. D) Strains from C) plus *mrc1Δ* (UPY1077) were synchronized as in B), and samples were assayed for Rad52-YFP foci as noted.

Appearance of immunofluorescent foci of γH2AX, a molecular species correlated with DNA damage-induced phosphorylation of a specific site on histone H2A, is a commonly used metric for DSB formation [34]. Correspondingly, during unchallenged asynchronous growth, very few wild-type cells (< 1%) demonstrate one or more γH2AX foci (Fig 2A). In contrast, addition of methyl methanesulfonate (MMS, known to cause DSBs [7]) results in γH2AX staining in the majority of the cells (Fig 2A). In striking contrast, the *mcm2DENQ* mutant exhibits significantly elevated levels of γH2AX foci during unchallenged growth (~25% of cells, Fig 2A), implying a strong tendency for DSB formation in this mutant.

We also examined the time of occurrence of γH2AX foci in the *mcm2DENQ* mutant by analysis of cells released from G1 arrest that proceed synchronously through the cell cycle as defined by a parallel FACS analysis (Fig 1A). In the wild-type strain, < 2% of the cells display γH2AX foci during any point in the cell cycle (Fig 2B black). In contrast in the *mcm2DENQ* mutant, γH2AX foci are observed at significant levels only after completion of bulk DNA replication: peak levels (~35%; Fig 2B red) are seen after 2C DNA content is achieved and prior to mitosis, as defined by restoration of 1C content (the period defined in budding yeast as "G2/M" (Fig 1A)).

#### Rad52 foci

Rad52 is specifically required for DSB repair and is able to bind both ss- and ds-DNA *in vitro* ([35, 36] and references therein). Moreover, Rad52 is a prominent component of cytologically-visible DNA DSB repair complexes [35, 37]. We used a YFP-fusion construct of Rad52 to ask whether the level of Rad52 foci is elevated during any stage of the cell cycle. In a wild-type strain, very few cells produce one or more fluorescent Rad52 foci during any stage of the cell cycle (~1%, Fig 2C and 2D black). In contrast, synchronized cultures of the *mcm2DENQ* mutant generated elevated levels of Rad52 foci with formation kinetics nearly identical to that observed for γH2AX foci (Fig 2C and 2D red). Moreover, over-expression of the *mcm2DENQ* allele did not reduce Rad52 foci levels, confirming that limiting levels of *mcm2DENQ* protein do not cause the observed DNA damage (S1A Fig). Further, we categorized cell cycle progression cytologically among single cells in an asynchronous population of *mcm2DENQ* by their budding index, a reliable measure of cell cycle progression in yeast [38]. Although the total population of cells in such a culture was well distributed throughout the cell cycle, the majority of cells that contain Rad52 foci were in G2/M (84%, S1BC Fig).

The simplest interpretation of these findings, taken together, is that in the *mcm2DENQ* mutant: (i) DNA damage arises after S-phase, likely during G2/M (see nocodazole experiments below), and (ii) this damage includes DSBs. This unusual phenotype has precedence in both budding yeast and in metazoan fragile sites (Discussion).

### DNA damage that arises in the *mcm2DENQ* mutant is distinct from that generated by stochastic replication fork collapse

Since Mcm2-7 is a core replication factor, DNA damage in the *mcm2DENQ* mutant could simply result from stochastic fork collapse during S-phase. However, the kinetics of damage foci appearance described above suggest that the explanation may lie elsewhere and, more specifically, might reflect formation of DSBs analogous to those observed in *mec1-4* and metazoan fragile sites (Discussion). We have explored these possibilities by comparing the *mcm2DENQ* mutant phenotypes side-by-side with those of *mrc1Δ*, which is known to give rise to structurally unstable replication forks that undergo spontaneous collapse in S-phase during unchallenged growth [8].

#### Timing of DNA damage during unchallenged growth

**Fig 3:**
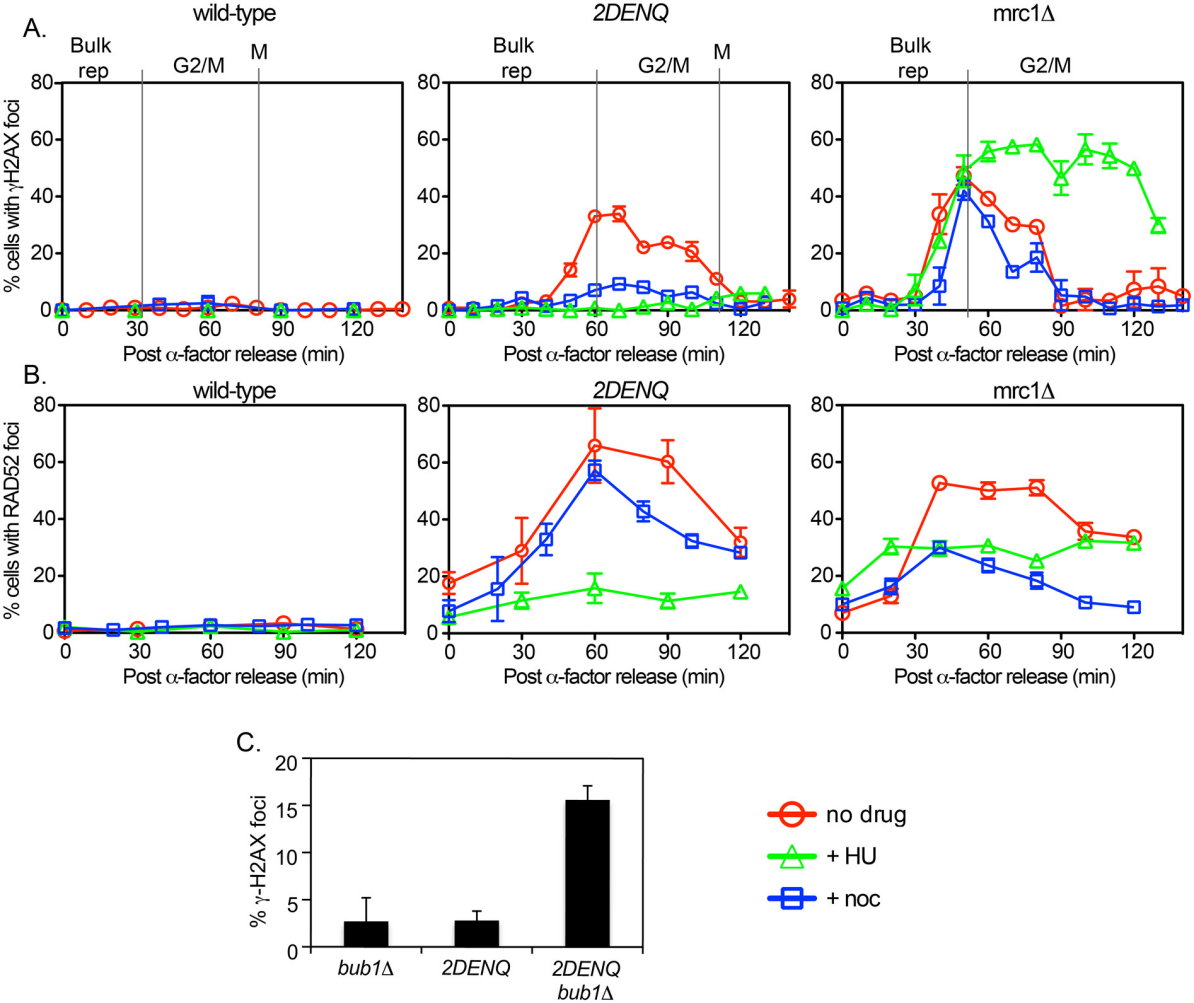
The effect of HU and nocodazole on *mcm2DENQ* DNA damage. A) Time-course analysis of γH2AX foci during the cell cycle. Indicated strains from 2B were synchronized, released into fresh YPD ± nocodazole (15 ug/ml) or HU (200 mM) as indicated, and samples were processed for γH2AX immunofluorescence. Data for the no drug timecourses were replotted from 2B. Cell cycle transitions noted are derived from a parallel FACS analysis of the indicated strains (Fig 1A). B) Time-course analysis of Rad52-YFP foci during the cell cycle. Indicated strains from 2D were synchronized and samples were assayed for Rad52-YFP foci after HU or nocodazole treatment as noted. Data for the no drug timecourses were replotted from 2D. C) γH2AX immunofluorescence of *bub1Δ* (UPY706), *mcm2DENQ* (UPY499), and *mcm2DENQ bub1Δ* (UPY707) + Nocodazole (2 hours) after release from α-factor.

In synchronized cultures of the *mrc1Δ* mutant, the level of γH2AX foci-positive cells dramatically increases and peaks at about the time of S-phase, earlier than the S/G2-phase timing observed for the *mcm2DENQ* mutant (Figs 2B and 3A). Moreover, the appearance of Rad52 foci in these mutants follows nearly identical kinetics to the development of γH2AX foci (Figs 2D and 3B). These results confirm the occurrence of fork collapse in the *mrc1Δ* mutant and support the view that the appearance of DNA damage foci in the *mcm2DENQ* mutant has a different cause.

#### Response to exogenous replication stress

In general, replication stress (*e*.*g*., HU) stimulates replication fork collapse and DSB formation [39, 40]. Correspondingly, mutations that compromise replication fork stability are particularly sensitive to HU and demonstrate an increase in stochastic fork collapse, accompanied by production of single-stranded DNA (ssDNA) that eventually leads to fork breakage [7, 41–44]. In a wild-type strain, exposure to HU has little effect: very few cells exhibit γH2AX foci at any time during the cell cycle, either without or with HU addition (<2% in both cases; Figs 2A and 3A), implying, importantly, that replication fork arrest *per se* does not in itself trigger this DNA damage signal.

**Fig 4:**
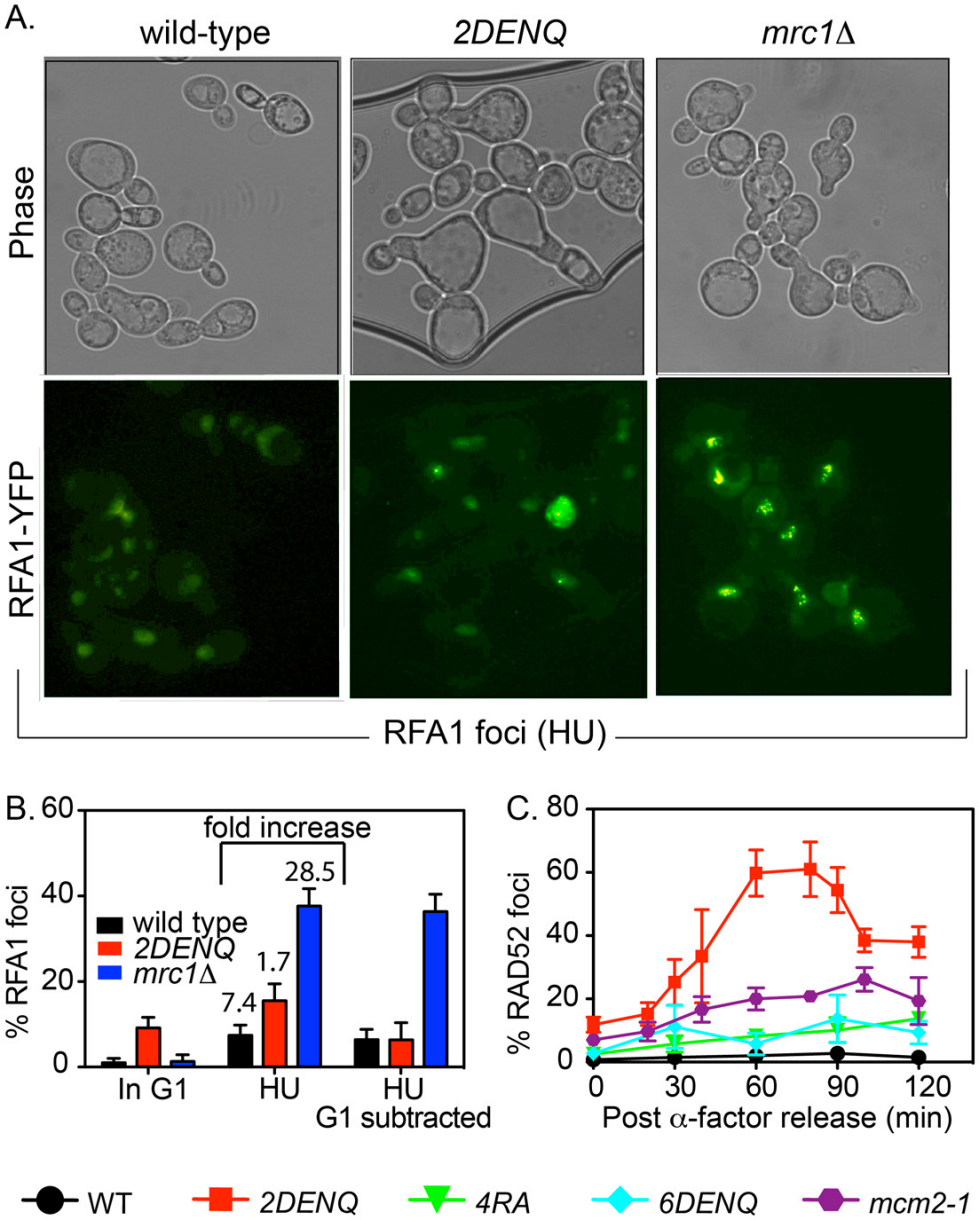
DNA damage *in mcm2DENQ* does not stem from stochastic fork collapse. A) Cultures of the wild-type (UPY1169), *mcm2DENQ* (UPY1168) and *mrc1Δ* (UPY1238) strains were synchronized with α-factor, released into fresh YPD + HU (200mM) for 120 minutes, and processed for phase contrast and Rfa1-YFP fluorescence (green). Representative micrographs are shown. B) Fraction of cells containing 1 or more Rfa1-YFP foci was quantified from cultures arrested in G1 (α-factor arrest), S-phase (120 minutes in HU), and S-phase data normalized for initial G1 Rfa-YFP levels. C) The indicated strains from 2C plus *mcm4RA* (UPY1022), *mcm6DENQ* (UPY1017), and *mcm2-1* (UPY1079) were grown and scored for Rad52 foci as in Fig 2D. Data from the *mcm2DENQ* strain were replotted from 2D.

In contrast, in the *mrc1Δ* mutant, HU greatly exacerbates an already pronounced occurrence of γH2AX foci seen in the absence of HU. Foci again rise during S-phase, but to an even higher level than in the absence of HU (Fig 3A). Moreover, the foci that occur in the presence of HU persist for an extended period of time relative to foci that arise during unchallenged replication (Fig 3A, compare red and green). The levels of Rad52 foci are similarly stabilized in the *mrc1Δ* mutant in the presence of HU. However, in this case HU does not stimulate production of additional DNA damage foci but instead partially suppresses their formation (Fig 3B), an observation consistent with the finding that HU slows DSB resectioning and reduces Rad52 foci levels under this condition [45]. The same patterns are also observed for Rfa1-YFP foci, a subunit of the DNA single-strand binding protein RPA that is commonly used as a cytological metric for ssDNA production (Fig 4A) [37]. Wild-type cells treated with HU exhibit a modest increase in the frequency of Rfa1 foci during S-phase (~ 7 fold increase, Fig 4B), while the *mrc1Δ* strain demonstrates a greatly enhanced level of Rfa1-YFP foci under these conditions (~ 28 fold increase, Fig 4B).

In sharp contrast, the formation of DNA damage foci in the *mcm2DENQ* mutant occurs very differently to replication stress; the presence of HU almost completely *suppresses* the formation of γH2AX and Rad52 foci rather than stimulates their production relative to their levels in G1 (Fig 3A and 3B). Moreover, HU causes little or no increase in the levels of Rfa1 foci in the *mcm2DENQ* mutant relative to those observed in G1 (p-value = 0.4, Fig 4B). Further, if the data is normalized to G1 Rfa1 foci levels, the wild-type and the *mcm2DENQ* mutant have indistinguishable Rfa1 foci levels in HU (p-value = 0.99, Fig 4B).

Although HU is an inhibitor of S-phase, it should be noted that substantial elongation (4–5 kb from all early origins in both the wild-type [46] and the *mcm2DENQ* mutant [25]) occurs under these conditions before fork progression wanes. In addition, reducing endogenous replication stress by increasing dNTP levels (by inclusion of the *sml1Δ* mutation [10]) is unable to significantly suppress γH2AX foci-formation in the *mcm2DENQ* mutant (Fig 2B). Thus, in contrast to the enhanced stochastic fork collapse observed in the *mrc1Δ* mutant, HU addition completely blocks development of DNA damage foci in the *mcm2DENQ* mutant.

### DNA damage in the *mcm2DENQ* mutant is different from that of other *mcm* mutant mutants

Several other viable ATPase active site alleles of Mcm2-7 are known. These include the *mcm4R701A* and *mcm6DENQ (DE638/39NQ)* substitution alleles of the Mcm 4/6 active site [25, 27, 47]. In addition, *mcm2-1* (*mcm2E392K*) is a hypomorphic allele isolated in the original genetic screen that identified Mcm2 (via defects in "mini-chromosome maintenance" [48]). All three mutants exhibit a higher level of Rad52 foci than wild-type. However, in contrast to the *mcm2DENQ* mutant, their levels were much lower and demonstrated no sharp peak of accumulation (Fig 4C). These data suggest that the defects conferred by the *mcm2DENQ* mutant are qualitatively and quantitatively different than those produced by other *mcm* mutants, consistent with our previous findings that the *mcm2DENQ* allele demonstrates other unique biological [25] and biochemical properties [27] not shared by these other alleles (Discussion).

### Occurrence of DNA damage in the *mcm2DENQ* mutant requires progression through G2/M

Since the *mcm2DENQ* mutant exhibits DNA damage in G2/M, we were curious to know whether formation of such damage requires spindle tension. To test this possibility, we examined the effect of the mitotic inhibitor nocodazole on γH2AX formation. Interestingly, we find that nocodazole blocks formation of γH2AX foci (but not Rad52 foci, see Discussion) in the *mcm2DENQ* mutant but not the *mrc1Δ* mutant (Fig 3A). However, nocodazole not only prevents spindle assembly via microtubule depolymerization but also, as a secondary consequence, activates the spindle assembly checkpoint (SAC) to trigger metaphase arrest. Thus, the effect of nocodazole on DNA damage in the *mcm2DENQ* mutant could reflect a requirement for progression through metaphase. To distinguish between these possibilities we asked whether the formation of DNA damage in *mcm2DENQ* is still blocked by nocodazole when cell cycle arrest is eliminated by loss of the SAC component Bub1 [49]. We find that when a *mcm2DENQ bub1* double mutant is treated with nocodazole, cell cycle progression occurs normally and γH2AX foci appear at levels similar to the corresponding untreated cells (Fig 3C), suggesting that DNA damage formation in the *mcm2DENQ* mutant is not a consequence of spindle tension, but rather depends upon cell cycle progression through G2. In addition, consistent with our results, several other studies have shown that spindle forces by themselves are insufficient to mechanically break mitotic chromosomes [50, 51]. Moreover, nocodazole treatment has no effect on the level of γH2AX foci exhibited by the *mrc1Δ* mutant (Fig 3A), as expected from the fact that they arise during S-phase due to replication fork collapse. These data provide strong further evidence that DNA damage foci occur in *mcm2DENQ* during G2/M rather than late S-phase.

### The *mcm2DENQ* mutant accumulates RNA:DNA hybrids

If the observed DNA damage in the *mcm2DENQ* mutant is not a consequence of spontaneous fork collapse, what causes its formation? Previous studies suggest that collisions between replication forks and active transcription units result in altered chromosome topology and the generation of RNA:DNA hybrid molecules (reviewed in [21]). In wild-type cells, this species accumulates only transiently; in contrast, such hybrids might occur at elevated levels in the *mcm2DENQ* mutant and cause genomic instability.

**Fig 5:**
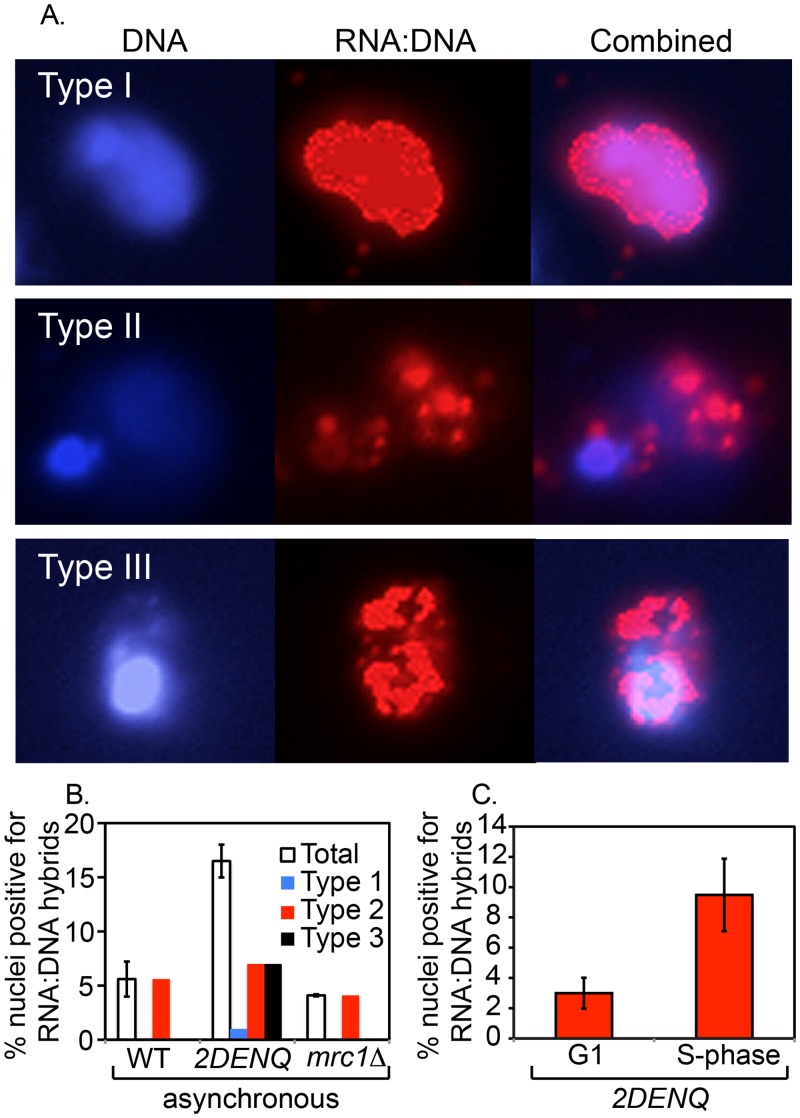
The *mcm2DENQ* mutant accumulates RNA:DNA hybrids. A) The mcm*2DENQ* mutant (UPY1014) was assayed as chromosome spreads for total DNA (DAPI, blue) and indirect immunofluorescence of RNA:DNA hybrids (red) (Materials and Methods). Differences in the co-localization of these signals allow assignment of individual cells into one of three distinguishable classes (representative types shown). B) Quantitation of both the level of each individual type of RNA:DNA hybrid as well as the sum (total) of all three types from asynchronous cultures of wild-type (UPY938), *mcm2DENQ* (UPY1014), and *mrc1Δ* (UPY1077). C) Total percent of all types of RNA:DNA hybrids from the *mcm2DENQ* mutant (UPY1014) arrested either in G1 (α-factor) or S-phase (200 mM HU).

To investigate this possibility, we examined formation of RNA:DNA hybrids in our strains used a previously-developed assay involving indirect immunofluorescence analysis of spread chromosomes [52]. The use of spread chromosomes, versus whole cells, has two advantages. First, it lowers the level of non-specific background staining (D. Koshland, per. com.). Second, as the nucleolus stains poorly with DAPI and physically separates from the rest of the nucleus in this procedure, one can specifically localize RNA:DNA hybrids with respect to either/both of these compartments ([53]). Specifically, as shown previously, three types of staining patterns can be defined (Fig 5A) [53]: The Type I pattern demonstrated a nearly total co-localization of the RNA:DNA hybrid staining and bright DAPI staining. The Type II pattern comprises RNA:DNA localization to a faint DAPI staining region adjacent to the main DAPI staining body. The Type III pattern corresponds to robust RNA:DNA hybrid localization to both the major and fainter DAPI staining regions. Type I, Type II and Type III patterns correspond, respectively, to localization of RNA:DNA hybrids to the nucleus only, to the nucleolus only, or to both compartments. Control experiments further show that the fraction of positively-staining nuclei is increased dramatically in a strain that eliminates several nucleases that specifically degrade RNA:DNA hybrids and that, oppositely, staining is virtually eliminated by treatment of spread preparations with commerically available RNaseH prior to incubation with antibody (S1D Fig).

In an asynchronous culture of the wild-type strain, ~6% of nuclei exhibit positive staining for RNA:DNA hybrids, essentially all of which are of Type II (nucleolar staining only, Fig 5B left). In striking contrast, in an asynchronous culture of the *mcm2DENQ* mutant, ~17% of nuclei exhibit staining which are equally distributed between Type II (7%, and thus similar to the level seen in the wild-type strain) and Type III (7%; staining in both compartments, which is thus specific to the *mcm2DENQ* mutant). Only a handful show nuclear staining only (Type I; 1%, Fig 5B middle). This pattern implies that RNA:DNA hybrids not only accumulate at elevated levels in the *mcm2DENQ* mutant but, in addition, form specifically in the nucleus. Importantly, parallel analysis further reveals that the pattern of RNA:DNA hybrid staining in *mrc1Δ* is essentially the same as in the wild-type strain (Fig 5B right).

To further define the time of appearance of RNA:DNA hybrids in the *mcm2DENQ* mutant, we examined cultures that were arrested either in G1 (by treatment with α-factor) or in early S-phase (by treatment with HU). The frequency of positively-staining nuclei is low in G1 arrest (3%) and much higher, ~9%, in HU-arrested cells. We infer that RNA:DNA hybrids arise during S-phase, not during G1 and not in post-S-phase stages (*e*.*g*., G2/M). The somewhat lower level seen in HU-arrest versus asynchronous cultures is not surprising since the latter will contain nuclei at all stages of S-phase rather than only early S-phase. As HU blocks the formation of DNA damage foci in the *mcm2DENQ* mutant (above), these results imply that RNA:DNA hybrids first accumulate at elevated levels during S-phase, an event that then leads to the development of DNA damage foci during G2/M via additional specific events (Discussion).

### The DNA damage phenotype of *mcm2DENQ* is suppressed by over-expression of either RNaseH or Topoisomerase 1

**Fig 6:**
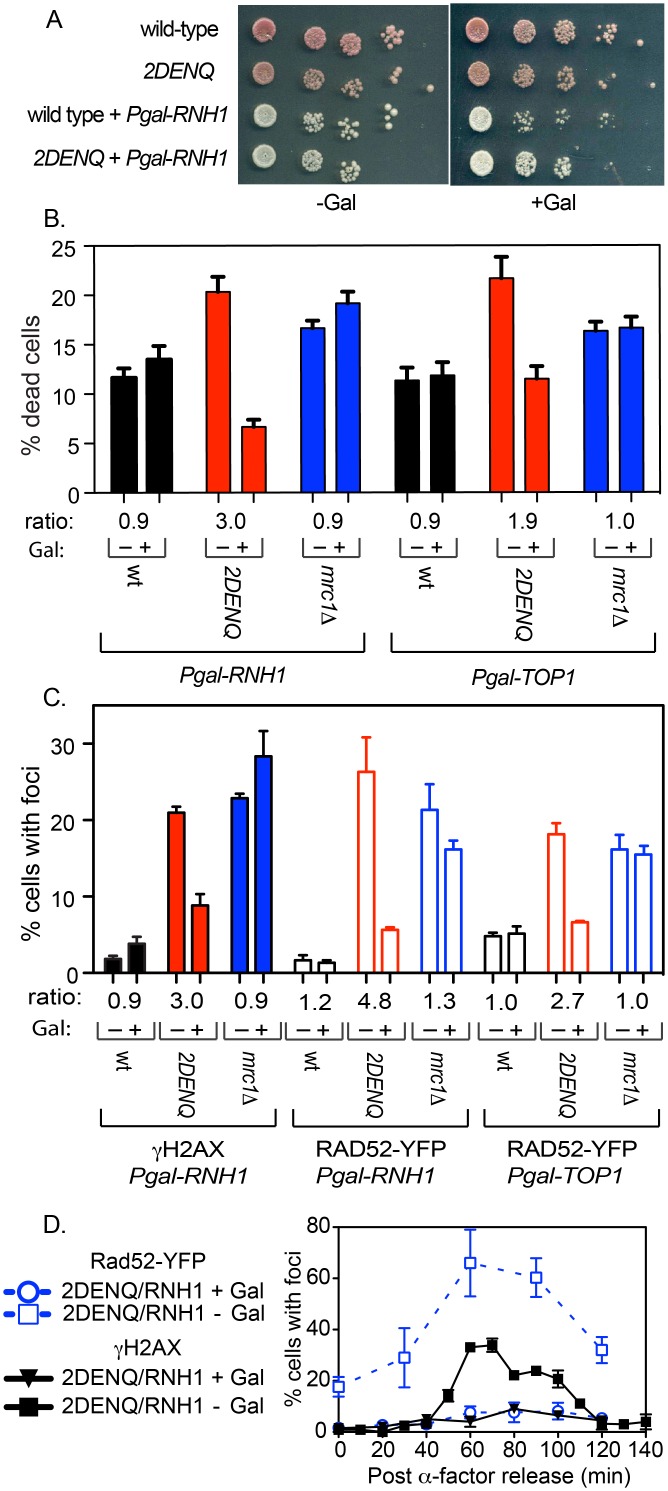
Over-expression of RNaseH and TopI suppress formation of DNA damage foci and cell death in the *mcm2DENQ* mutant. A) Plate assay of strains carrying RNaseH over-expression vectors. Strains ± P_gal_-RNH1over-expression plasmid (pUP1230) were tested on rich media under non-induced (-Gal) or induced (+Gal) conditions. Strains tested were wild-type without plasmid (UPY938) or with *RNH1*-expressing plasmid (UPY1289), *mcm2DENQ* without plasmid (UPY1014), or with *RNH1*-expressing plasmid (UPY1290). B) Cell death as a function of either Rnh1 or Top1 over-expression. Assays were conducted as in Fig 1B, except the strains contained the indicated over-expression plasmid. Asynchronous cultures of the indicated strains were grown with either glucose (-Gal) or galactose (+Gal), and apoptotic cells were counted. Strains with the *RNH1* plasmid were wild-type (UPY1336), *mcm2DENQ* (UPY1337), *mrc1Δ* (UPY1338), while strains with the *TOP1* plasmid were wild-type (UPY1339), *mcm2DENQ* (UPY1340), *mrc1Δ* (UPY1341). C) Bar graph showing levels of cells containing γH2AX and Rad52-YFP foci following over-expression of either Rnh1 or Top1. Asynchronous cultures of the indicated strains were grown with either raffinose (-Gal) or galactose (+Gal), and cells containing γH2AX or Rad52-YFP foci were counted. Strains with the *RNH1* plasmid were wild-type (UPY1289), *mcm2DENQ* (UPY1290), *mrc1Δ* (UPY1304) and were assayed for both γH2AX and Rad52 foci, and strains containing the *TOP1* plasmid include wild-type (UPY1342), *mcm2DENQ (UPY1343)*, and *mrc1Δ* (UPY1344). D) Time course experiment examining γH2AX and Rad52-YFP foci in a *mcm2DENQ* strain (UPY1290) in the presence (+Gal) and absence (-Gal, growth in raffinose) of RNaseH1 over-expression. To initially maintain the *RNH1* expression plasmid, the culture was grown in selective media containing either 2% raffinose or 2% galactose for four hours; cells were subsequently transferred to rich media containing either 2% raffinose or 2% galactose for α-factor arrest and subsequent timecourse analysis.

We next examined the possibility that formation of RNA:DNA hybrids or altered DNA topology might contribute to the various *mcm2DENQ* phenotypes. Toward this end, we expressed either the *S*. *cerevisiae RNH1* (RNaseH1) or *TOP1* (Topoisomerase 1) genes from the strongly inducible *GAL1* promoter on a high-copy plasmid [54–56]. Rnh1 over-expression has been previously shown to reduce cellular levels of such hybrids in yeast [53, 54]. Top1 enzymatically removes both positive and negative supercoils from DNA during replication [56]. We verified that over-expression of either protein did not substantially interfere with cell growth or viability (Fig 6A), and that growth with galactose does not substantially affect the level of Rad52 foci in the parental strains lacking the test plasmids (S1E Fig).

We first tested the ability of *RNH1* or *TOP1* to suppress the viability defect of the *mcm2DENQ* mutant in asynchronous cultures. Over-expression of these proteins had no significant effect on the level of cell death in wild-type (p-value > 0.29) but, in contrast, resulted in a 2–3 fold suppression of cell death in the *mcm2DENQ* mutant (p-values < 0.0025, Fig 5B). These results strongly suggest that formation of RNA:DNA hybrids is involved in the increased level of *mcm2DENQ*-dependent cell death.

The effect of RNaseH on γH2AX foci formation was similarly tested. Rnh1 over-expression has little effect on the low basal of γH2AX foci seen in wild-type cells (Fig 6C). In contrast, Rnh1 over-expression in *mcm2DENQ* cells resulted in a significant decrease in the incidence of γH2AX foci (2.4-fold, p-value = 0.0025, Fig 6C).

We next examined the ability of RNaseH and Top1 over-expression to suppress Rad52 foci development. In the wild-type strain, over-expression of either *RNH1* or *TOP1* had little effect on the formation of Rad52 foci (1.0–1.2 fold, p-values >0.38) (Fig 6C). In sharp contrast, over-expression of either protein in the *mcm2DENQ* mutant caused a marked reduction of Rad52 foci levels with both *RNH1* (4.8 fold, p-value = 0.037) and *TOP1* (2.7 fold, p-value <0.0001) (Fig 6C). These results strongly suggest that formation of Rad52 foci in *mcm2DENQ* depends upon RNA:DNA hybrids.

We further asked if the Rnh1-mediated suppression of DNA damage in *mcm2DENQ* also occurs in a cell cycle-specific fashion. We find that over-expression of *RNH1* (+Gal) in synchronized *mcm2DENQ* cells dramatically suppresses the formation of both Rad52 and γH2AX foci specifically during S-phase relative to the control (-Gal, Fig 6D). These findings provide additional evidence that the *mcm2DENQ* mutant possesses a primary defect in DNA replication that manifests itself only later in the cell cycle as DNA damage (*e*.*g*., DSBs).

Finally, in accord with the observation that the *mrc1Δ* mutant does not show elevated levels of RNA:DNA hybrids, *RNH1* over-expression has no statistically significant effect on the *mrc1Δ* mutant with respect to either cell death (p-value >0.1; Fig 6B) or γH2AX foci levels (p-values > 0.12, Fig 6C). Moreover, over-expression of either *RNH1* or *TOP1* is unable to suppress the formation of Rad52 foci in *mrc1Δ* (Fig 6C). These findings further highlight the unique nature of the *mcm2DENQ* DNA damage defects as compared to the *mrc1Δ* mutant.

Taken together, these results strongly indicate that the DNA damage phenotype in the *mcm2DENQ* mutant has its basis in altered DNA topology, which includes (but is not restricted to) the abnormal accumulation of stable RNA:DNA hybrids.

## Discussion

We have previously described an allele of the Mcm2-7 replicative helicase (*mcm2DENQ*) that has both unique biochemical properties [27, 28] and a unique role in the DRC cascade in response to externally-provided “replication stress” (*i*.*e*., HU treatment) [25]. Here, we provide evidence that this mutant is also involved in modulating DNA replication during unchallenged growth, and exhibits unusual replication-dependent DNA damage that is likely linked to an increased frequency of both apoptotic cell death and spontaneous genome rearrangement under this condition. Interestingly, as detailed below, these defects are strikingly similar to those observed previously in a unique allele of yeast *Mec1* (*ATR*) and to effects implicated in the formation of common fragile sites in mammalian cells. These observations, in combination with others, suggest that both Mcm2-7 and Mec1/ATR may coordinately participate in a novel replication “surveillance system” that both promotes normal elongation and prevents certain types of DNA damage.

### An unexpected link between Mcm2-7 and genome stability

Our investigation of the *mcm2DENQ* mutant reveals an interesting and unexpected array of DNA damage phenotypes.

First, we observe an abnormal increase of RNA:DNA hybrids in this mutant (Fig 5). This species appears during an aberrantly prolonged S-phase, and appears to largely target DNA in the nucleus rather than nucleolus, strongly suggesting that active replication leads to its accumulation. Moreover, it does not appear to be linked to stochastic fork collapse, as its formation is not stimulated in the *mrc1Δ* mutant. These data suggest that RNA:DNA hybrids are an aberrant consequence of altered DNA replication in the *mcm2DENQ* mutant.

Second, we observe after completion of bulk DNA replication the development γH2AX and Rad52 foci (Fig 2), both of which are canonically taken as markers for formation and repair of DSBs [34, 57], suggesting that *mcm2DENQ* exhibits DSBs. Moreover, the formation of such foci does not occur spontaneously but specifically requires passage through the spindle assembly checkpoint (SAC), but not the actual occurrence of mitotic chromosome segregation and/or spindle tension (Fig 3).

These two defects appear to be causally linked. 1) As RNA:DNA lesions occur in S-phase (HU arrest) much earlier than DNA damage foci, RNA:DNA hybrids are possible precursors of DNA damage foci in G2/M; 2) *In vivo* over-expression of either RNase H or Top1 almost completely eliminates both DNA damage foci (Fig 6) and elevated levels of cell death. Over-expression of either enzyme to suppress DNA damage foci likely involves loss of RNA:DNA hybrids, as aberrant DNA topology, presumably rectified by excess TOP1, drives RNA:DNA hybrid formation [58–60]. These observations provide additional evidence that accumulation of abnormal levels of RNA:DNA hybrids is the primary cellular defect in this mutant, while DNA damage foci are a secondary manifestation. Thus taken together, these data suggest that a specific DNA replication defect in the *mcm2DENQ* mutant leads to formation of RNA:DNA hybrids during S-phase, these in turn persist until the cells pass through the spindle assembly checkpoint. For currently unknown reasons, some fraction of the RNA:DNA hybrids are converted into DSBs at this point, which in turn presumably leads to a reduction in cellular viability as the cells attempt to traverse M phase with damaged DNA.

We acknowledge that there are several caveats and limitations to this proposed scenario. For example, nocodazole does not block formation of Rad52 foci in the *mcm2DENQ* mutant (Fig 3B), but does partially suppress Rad52 foci in the *mrc1Δ* mutant (Fig 3B) We speculate that γH2AX and Rad52 foci might be identifying somewhat different DNA damage intermediates. DSBs have been most closely associated with γH2AX foci [61], although γH2AX has been shown to form in response to other stimuli in higher organisms [62]. In contrast, Rad52 foci may serve as a broad metric for an array of different DNA damage types that include DSBs. As Rad52 is involved in both ss and ds-DNA mediated processes [61, 63–67], we propose that Rad52 binds some species (*e*.*g*., RNA/DNA hybrids) that act as a precursor to *bona fide* DSBs. Moreover, nocodazole has a variety of additional biological effects distinct from its well-known role in preventing microtubule formation [68]. In addition, although we favor the possibility that aberrant replication stimulates the formation of both DNA damage species, we cannot disprove the possibility that the *mcm2DENQ* mutant instead disrupts DNA repair and prevents timely removal of pre-existing lesions. Finally, DNA replication may not completely finish prior to M phase in this mutant, a possibilty that might additionally complicate our model. However, a side-by-side comparison of the *mcm2DENQ* DNA damage phenotype with that of *mrc1Δ* implies specifically that the S-phase lesions that accumulate in *mcm2DENQ* do not induce spontaneous fork collapse. Thus, whatever the ultimate mechanism behind G2/M DNA damage in the *mcm2DEN*Q mutant, this phenotype is novel and likely reflects a currently unknown regulatory defect during DNA replication.

### RNA/DNA hybrids and unresolved conflicts between replication forks and transcription complexes

The precise S-phase events that trigger accumulation of RNA:DNA hybrids in the *mcm2DENQ* mutant remain to be defined. However, an obvious and intriguing candidate would be conflicts between opposing replication forks and transcription complexes. RNaseH-suppressible RNA:DNA hybrids are known to accumulate during impeded transcription of any of the three eukaryotic RNA polymerases [59, 69, 70]. Such hybrids are believed to arise from either of two mechanisms: 1) Occlusion of factors that normally block formation of RNA:DNA hybrids (*e*.*g*., including members of the TREX/THO complexes that aid mRNA export [71, 72], and/or, 2) Altered DNA supercoiling formed in the wake of a blocked transcription complex when encountered by a converging replication fork [21, 73]. We strongly favor the second possibility because aberrant topology at collision sites is known to trigger a relevant regulatory surveillance response ([74], below), and over-expression of Top1, an enzyme that relaxes supercoiling, suppresses most of the deleterious phenotypes of the *mcm2DENQ* mutant (Fig 6B and 6C). Moreover, this particular phenotype appears separable from canonical DRC function, as the DRC mutant *mrc1Δ*, that demonstrates elongation defects [75], retains wild-type levels of RNA:DNA hybrids (Fig 6B and 6C).

In principle, the RNA:DNA hybrids that form in the *mcm2DENQ* mutant could arise as very short unprocessed Okazaki fragments that still contain the ~8 bp RNA primer leftover from lagging strand DNA synthesis. However, cells have multiply redundant mechanisms to process Okazaki fragments following fork passage [76], and defects in these processes have not yet been observed to generate RNA:DNA hybrids as operationally defined by us and others (*e*.*g*., [23]). Moreover, as Okazaki fragments are a normal and essential feature of DNA replication and remain stably bound to the DNA template prior to processing, it is unclear why exposure to HU or nocodazole would block or reduce their processing into DSBs. These observations argue against a role for defective Okazaki-fragment processing in the elevated levels of RNA:DNA hybrids in the *mcm2DENQ* mutant.

Given the above, we favor the hypothesis that the observed accumulation of RNA:DNA hybrids in the *mcm2DENQ* mutant represents unresolved conflicts between replication forks and transcription complexes. Determining whether such hybrids accumulate at all highly transcribed regions or at only a few specific sites will require future genomic analysis. Moreover, whether the hybrids are in fact generated by the conflict or are instead preexisting and provide a stable impediment to elongation cannot be currently distinguished by our data.

### Resolution of replication/transcription conflicts via ATR/Mec1-mediated opening of the Mcm gate

The six distinct MCM active sites do not contribute equally to replication; some sites appear dedicated to DNA unwinding, while the others have a likely regulatory role (reviewed in [26]). At least one function of these regulatory ATPase active sites is to modulate the conformation of a reversible discontinuity between two specific subunits (the Mcm2/5 gate, [47, 77]). Modulation of this gate is essential to Mcm2-7 function: evidence indicates that gate closure is required for both initial DNA loading at replication origins [78], and subsequent helicase activity [47, 79]. Previous biochemical investigation has shown that the *mcm2DENQ* allele has no effect on Mcm2-7 helicase activity but does cause a specific defect in the opening of the Mcm2/5 gate [27]. This biochemical property is unique among a vast array of other Mcm ATPase alleles studied ([27, 47]). It is tempting to speculate that the *mcm2DENQ* mutant defects *in vivo* directly stem from this biochemical defect.

**Fig 7:**
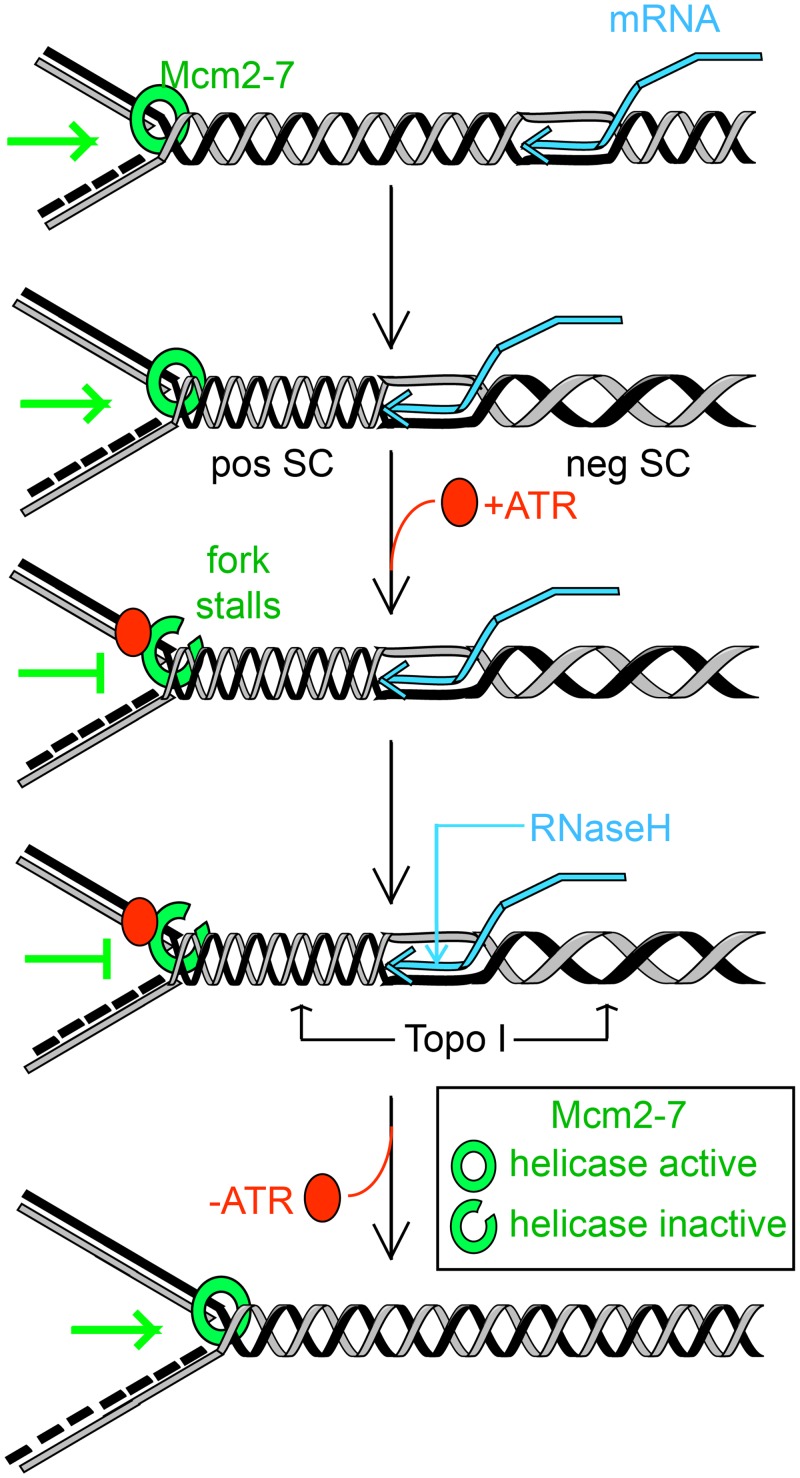
Proposed model for Mcm2-7/ATR DNA damage surveillance. In wild-type cells, individual elongating replication forks pause in an ATR-dependent manner when encountering, transcription bubble via modulation of the Mcm ‘gate’. Such transient pausing provides a temporal window to allow removal of topological perturbations (putatively via Top1 and RNaseH). However, in the *mcm2DENQ* mutant, failure to regulate gate opening aggravates the accumulation of supercoiling that both stabilizes RNA:DNA hybrids and leads to subsequent DNA damage. Pos SC- positive supercoiling, neg SC- negative supercoiling.

Based on the above, we propose the following basic model (Fig 7). In wild-type cells, a head-on conflict between a DNA replication fork and a transcription elongation complexes triggers opening of the Mcm2/5 gate, thereby blocking helicase activation and causing the replication fork to arrest. Mcm2-7 would nonetheless remain associated with the replication complex. Once the conflict is resolved (presumably by release of the transcription complex and removal of any RNA:DNA hybrids and aberrant DNA topology), the gate would close and DNA replication would resume (Fig 7). In the *mcm2DENQ* mutant, a reduction or inability to open the Mcm 2/5 gate and stop elongation would then prevent release of a nascent transcript, which eventually results in DNA damage detectable as either γH2AX or Rad52 foci (below). Indeed, such head-on collisions are known to be recombinogenic (transcription-associated recombination (TAR), reviewed in [24]) which might explain the distinctive appearance of the various damage foci in our mutant. Interestingly, both RNaseH and Top1 activity are implied in constraining TAR [24]. However, a direct test of these hypotheses is beyond the scope of this work.

The question then arises: what signal triggers gate opening? Previous studies have shown that replication-transcription conflicts trigger an ATR/Mec1-mediated surveillance response, which mediates collision resolution [80–82]. The triggering signal appears to be topological, *e*.*g*., hyper-accumulation of positive supercoiling in the convergence region [74]. Thus, ATR/Mec1 could modulate the DNA unwinding activity of Mcm2-7 as part of this surveillance response. This possibility fits well with our previous findings that Mcm2-7 participates in the ATR-mediated checkpoint response to HU-induced replication stress, downstream of ATR activation, with the *mcm2DEN*Q mutation conferring a specific defect in this process [25].

Moreover, this possibility if supported by observations of a specialized allele of *MEC1/ATR*, *mec1-4*. In the *mec1-4*/*atr* mutant, DSBs (directly observed by physical assay) occur at G2/M as a delayed response to a problem that arises in S-phase, specifically an inability of replication forks to progress through certain genetically-defined "slow zones" [15] that in general have been shown to correlate with highly transcribed genes [83]. In both the *mec1-4* and *mcm2DENQ* mutants, DSBs/DNA damage is suppressed by HU treatment [84]. Furthermore, DSB formation does not require spindle tension but does require progression through G2/M as defined by the same criteria applied above to *mcm2DENQ* [84, 85]. Moreover, in the case of the *mec1-4* mutant, alleles of *top2* and the condensins have been shown to suppress DSB formation [85], in accord with the important role that G2/M DNA topology likely plays in DSB formation. However, the detailed relationship between these two alleles remains to be defined, such as whether *mec1-4* alleles accumulate RNA:DNA hybrids or if *mcm2DENQ* phenotypes involve replication slow zones.

Together, these findings point to a close analogy between the *mec1-4* and *mcm2DENQ* mutations. By implication, the current findings now suggest that: (i) *mcm2DENQ* may have a cryptic defect in DNA replication whose effects are manifested later, during progression through G2/M; and (ii) the DNA damage in *mcm2DENQ* is indeed likely to include DSBs, as suggested above. These analogies further raise the possibility that there could be a direct link between the roles of ATR and Mcm2-7 in ensuring regular DNA replication during S-phase.

### Relationship among Mcm2-7, ATR/Mec1, and common fragile sites

The DNA damage observed in the *mcm2DENQ* and *mec1-4* mutants have characteristics reminiscent of common fragile site breaks (CFS) observed in metazoans (reviewed in [86]): 1) CFSs usually map to regions that undergo late DNA replication, implying that the breaks themselves likely occur after completion of bulk DNA replication; 2) The formation of CFSs is greatly stimulated by either loss or inactivation of ATR/Mec1, or conditions that hamper replication fork progression; (3) Metazoan fragile sites localize to specific chromosome regions that often correlate with extremely long coding regions; and 4) CFSs are often sites of RNA:DNA hybrid accumulation [81]. Thus, the commonalities between CFS breaks and G2/M breaks strongly suggest that they are manifestations of the same Mcm2-7-dependent phenomena.

Although much is known about CFSs, the complex events surrounding their formation has hampered studies to determine their mechanism. Our work, in combination with other studies of metazoan fragile sites (*e*.*g*., [81]), strongly suggest that in fact many or most CFS breaks emanate from an inability to resolve fork conflicts, rather than from a simple inability to finish elongation, with the Mcm2-7 complex possibly playing an important role in these events.

## Materials and Methods

### Yeast methods

*S*. *cerevisiae* strains and plasmids are listed in Tables A and B in S1 data. Unless otherwise noted, all strains are isogenic derivatives of *W303* and were constructed using standard yeast methodology (details are available upon request); cultures were grown at 30°C; cell cycle synchronization, arrest, and FACS analyses were carried out as previously described [25]. Nocodazole was used at 15μg/ml and hydroxyurea was used at 200mM.

### Cell death

This assay was performed as described [87]. Briefly, asynchronous cells in log phase were incubated with 10μg/ml dihydrodichloro-fluorescein diacetate (D6883 (Sigma) from 2.5mg/ml stock prepared in 100% ethanol) for 2 hours at 30°C in the dark with gentle mixing. After incubation, cells were spotted on polylysine-coated slides and immediately visualized using a Zeiss Axioskop 40 microscope equipped with a green filter set (Zeiss filter set #38) and a CCD camera for image acquisition. >100 cells were counted for each sample.

### Gross chromosomal rearrangement

The GCR assay was performed as described [31]. Briefly, a single colony was inoculated into 2 ml of YPD, grown overnight at 30°C, then subcultured the following day into 50ml of YPD media. The 50ml culture was grown overnight to saturation (~1X10^8^ cells/ml). An aliquot was withdrawn, diluted, sonicated and spread onto YPD plate to access viable count. The culture was concentrated as needed by centrifugation and spread onto synthetic media lacking arginine and containing 5-fluoroorotic acid (FOA, 1mg/ml) and L-Canavanine (CAN, 60μg/ml) at roughly 4X10^8^ cells/plate to assay the level of translocations. After 4 days of incubation at 30°C, double-resistant (FOA^R^ CAN^R^) colonies were counted. GCR rate was calculated as previously described [31]. Results reported were the average ≥ 3 independent assays.

### DNA damage foci analysis

Assays to visualize γH2AX [34] or Rad52-YFP and Rfa1-YFP foci [35] were conducted as described. The polyclonal antibody to γH2AX was a kind gift from William Bonner (NCI). Slides were viewed under the Zeiss Axioskop 40 microscope, and ≥ 100 cells were counted for each sample; the fraction of cells containing one or more foci were tabulated. Images were acquired using a mounted CCD-camera and processed with Axio Vision software. For RNaseH or Top1 over-expression experiments, cultures were grown overnight in YP + 2% raffinose, 0.1% glucose and induced by the addition of 2% galactose the subsequent day for 4–6 hours at 30°C prior to sample processing.

### Budding analysis of Rad52-YFP foci

Asynchronous culture of UPY1014 (*mcm2DENQ*) was grown to mid log-phase in rich media, cells were stained with Hoechst 33342 (5 ug/ml), and the budding index, prevalence of Rad52 foci, and DNA content were assayed in individual cells by fluorescence microscopy and tabulated.

### Chromosome spreads

The assay was conducted as described in [52]. Spreads were incubated in 1X PBS (Phosphate-buffered saline) for 30 minutes, blocked in 5% BSA, 0.2% non-fat dry milk in 1X PBS for 15 minutes and subsequently probed for RNA:DNA hybrids using the S9.6 antibody (1mg/ml, kind gift from D. Koshland) at a 1:500 concentration overnight at room temperature. The slides were washed in 1X PBS the subsequent day and incubated with goat anti-mouse Alexa-fluor 568 (1:1000 dilution, Life Technologies) for an hour in dark. Thereafter, slides were washed, mounted with Slowfade antifade containing 4',6-diamidino-2-phenylindole (DAPI, Life Technologies) and imaged with a Zeiss AxioObserver Z1 inverted fluorescence microscope with a mounted CCD camera using a 63X objective. Images were taken with the appropriate filter sets to separately record DAPI and RNA:DNA signal fluorescence; Adobe Photoshop was then used to colorize and combine the two images to facilitate their classification into one of the three types shown in Fig 5A.

### Statistical methods

Except as noted, all listed results represent the average and standard error of the means of at least three independent experiments. Unpaired student’s t-tests were used to calculate p-values of the various assays presented.

## Supporting Information

S1 Fig A) Over-expression of the *mcm2DENQ* allele does not suppress formation of Rad52 foci. A *mcm2DENQ* strain containing an integrated plasmid with an additional copy of the *mcm2DENQ* allele under the inducible *GAL1* promoter (UPY1284) was scored for Rad52 foci during asynchronous growth in either the presence (+ Gal) or absence (- Gal, growth in raffinose) of galactose. B) Bud distribution of *mcm2DENQ* cells containing Rad52-YFP foci. Results from a representative experiment of *mcm2DENQ* are shown. White bars–fraction of cells in the population that demonstrated the indicated budding index (N = 122). Blue bars–among cells that contained Rad52 foci, the fraction that demonstrate the indicated budding index (N = 100). Small budded cells (sm bud) were visually judged to have buds ~ 25% or less the size of the mother cell; medium budded cells (med bud) were judged to contain buds between 25–50% the size of the mother cell, and large budded cells (lg bud) contained buds between 50–100% the size of the mother cell. Cells in medial nuclear division (med. nuc. div.) had 2 nearly equal sized buds with genomic DNA in the neck region between cells. Telophase cells (telo) contained connected buds with clearly defined nuclei in each bud. Prior analysis indicates that cells with medium and large buds, medial nuclear division, and telophase are all in G2/M [38]. C) Representative examples of cells from A) that contain Rad52 foci. White = DNA, red = Rad52 foci. D) RNA:DNA hybrids are sensitive to exogenously added RNase H. A strain previously demonstrated to generate high levels of RNA:DNA hybrids (*rnh1Δ rnh201Δ* (KO175)) was assayed for RNA:DNA hybrids both in the absence (left) and presence (right) of 10 U of added RNase H (NEB M0297S). Sum (total) levels of all three types of RNA:DNA hybrids shown. E) Rad52-YFP foci in asynchronous cultures of wild-type (UPY938) or *mcm2DENQ* (UPY1014) and *mrc1Δ* (UPY1077). Cells were grown in rich media with either raffinose (blue bars) or galactose (white bars).
(TIF)

S1 Data Supporting tables. (DOCX)
