# Supplementary figures and images for: A Checkpoint-Related Function of the MCM Replicative Helicase Is Required to Avert Accumulation of RNA:DNA Hybrids during S-phase and Ensuing DSBs during G2/M
Source: PLoS Genet. 2016 Aug 24;12(8):e1006277. doi: 10.1371/journal.pgen.1006277 (PMC4996524; doi:10.1371/journal.pgen.1006277)

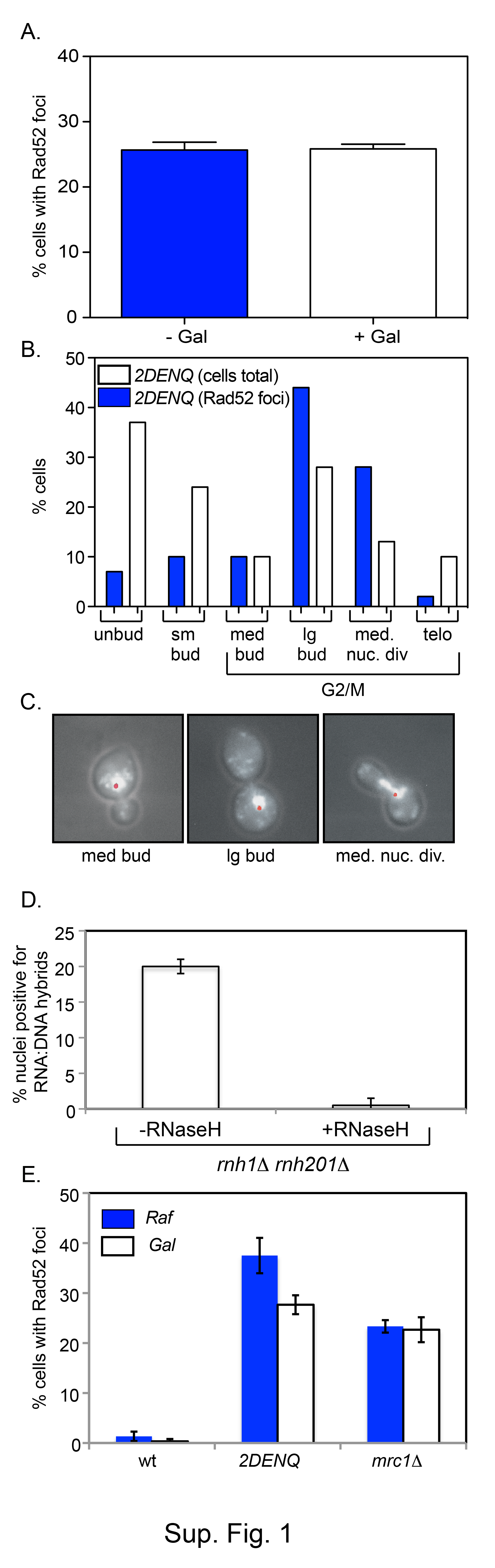

Supplement: S1 Fig — A) Over-expression of the mcm2DENQ allele does not suppress formation of Rad52 foci. A mcm2DENQ strain containing an integrated plasmid with an additional copy of the mcm2DENQ allele under the inducible GAL1 promoter (UPY1284) was scored for Rad52 foci during asynchronous growth in either the presence (+ Gal) or absence (- Gal, growth in raffinose) of galactose. B) Bud distribution of mcm2DENQ cells containing Rad52-YFP foci. Results from a representative experiment of mcm2DENQ are shown. White bars–fraction of cells in the population that demonstrated the indicated budding index (N = 122). Blue bars–among cells that contained Rad52 foci, the fraction that demonstrate the indicated budding index (N = 100). Small budded cells (sm bud) were visually judged to have buds ~ 25% or less the size of the mother cell; medium budded cells (med bud) were judged to contain buds between 25–50% the size of the mother cell, and large budded cells (lg bud) contained buds between 50–100% the size of the mother cell. Cells in medial nuclear division (med. nuc. div.) had 2 nearly equal sized buds with genomic DNA in the neck region between cells. Telophase cells (telo) contained connected buds with clearly defined nuclei in each bud. Prior analysis indicates that cells with medium and large buds, medial nuclear division, and telophase are all in G2/M [38]. C) Representative examples of cells from A) that contain Rad52 foci. White = DNA, red = Rad52 foci. D) RNA:DNA hybrids are sensitive to exogenously added RNase H. A strain previously demonstrated to generate high levels of RNA:DNA hybrids (rnh1Δ rnh201Δ (KO175)) was assayed for RNA:DNA hybrids both in the absence (left) and presence (right) of 10 U of added RNase H (NEB M0297S). Sum (total) levels of all three types of RNA:DNA hybrids shown. E) Rad52-YFP foci in asynchronous cultures of wild-type (UPY938) or mcm2DENQ (UPY1014) and mrc1Δ (UPY1077). Cells were grown in rich media with either raffinose (blue bars) or galactose (white b [file pgen.1006277.s001.tif]
