# Supplementary material for: A Checkpoint-Related Function of the MCM Replicative Helicase Is Required to Avert Accumulation of RNA:DNA Hybrids during S-phase and Ensuing DSBs during G2/M
Source: PLoS Genet. 2016 Aug 24;12(8):e1006277. doi: 10.1371/journal.pgen.1006277 (PMC4996524; doi:10.1371/journal.pgen.1006277)
Supplement: S1 Data — (DOCX) [file pgen.1006277.s002.docx]

Table A – *S. cerevisiae* strain list

| Strain | Relevant Genotype | Source |
| --- | --- | --- |
| W303A | *MATA, ade2-1, ura3-1, his3-11,15, leu2-3,112, can-100, trp1-1* | SP Bell |
| W303α | *MATa, ade2-1, ura3-1, his3-11,15, leu2-3,112, can1-100, trp1-1* | SP Bell |
| K0175 | *W303A +rnh201::loxP-HYGMX-loxP, rnh1::loxP-MATMX-loxP, chrIV1495420::loxP-KlURA3-loxP* | S. Jinks-Robertson [1] |
| UPY464 | *W303A + bar1::hisG* | This study |
| UPY499 | *W303A + bar1::hisG, mcm2DENQ* | This study |
| UPY622 | *MATa, ura3-52, leu2Δ1, trp1Δ63, his3Δ200, lys2ΔBgl, hom3-10, ade2Δ1, ade8, yel069::URA3 (S288C background)* | RDKY3615, R. Kolodner [2] |
| UPY687 | UPY622 + *mcm2DENQ-NatMX4* | This study |
| UPY694 | UPY622 + *rad50∆::loxP-his5+-loxP* | This study |
| UPY698 | UPY622 + *mrc1∆::loxP-his5+-loxP* | This study |
| UPY700 | *W303A + bar1::hisG, ade3∆::hisG, mrc1∆::loxP-URA3-loxP* | This study |
| UPY706 | *W303A +bar1::hisG, bub1∆::his5-lox* | This study |
| UPY707 | *W303A +bar1::hisG, bub1∆::his5^+^-lox, mcm2DENQ* | This study |
| UPY713 | *W303A + bar1::hisG, mrc1∆::loxP-his5^+^-loxP* | This study |
| UPY715 | *W303A + bar1::hisG, rad9∆::loxP-his5^+^-loxP, sml1∆::loxP-his5^+^-loxP, mrc1∆::loxP* | This study |
| UPY756 | *W303A + mcm2-1* | M358, M. Weinreich |
| UPY769 | *W303A + bar1::hisG,* *mcm2-1* | This study |
| UPY938 | *W303A + bar1::LEU2, RAD52-YFP* | W3749, K. Bernstein |
| UPY948 | *W303A + bar1::hisG, sml1∆::loxP-his5^+^-loxP, mcm2DENQ* | *This study* |
| UPY1014 | *W303A + bar1::LEU, RAD52-YFP, mcm2DENQ* | This study |
| UPY1017 | *W303A + bar1::LEU2, mcm6DENQ* | This study |
| UPY1022 | *W303A + lys2::hisG, RAD52-YFP, mcm4RA* | This study |
| UPY1077 | *W303A + bar1:LEU2, RAD52-YFP, mrc1∆::loxP-his5^+^-loxP* | This study |
| UPY1079 | *W303A + bar1::LEU2, RAD52-YFP, mcm2-1* | This study |
| UPY1140 | W303α + *bar1::LEU2*, *lys2∆*, *RFA1-YFP* | K. Bernstein |
| UPY1168 | *W303A + bar1∷hisG, RFA1-YFP, mcm2DENQ* | This study |
| UPY1169 | *W303A + bar1::LEU2,* *RFA1-YFP* | This study |
| UPY1238 | *W303A + bar1::LEU2, RFA1-YFP, mrc1∆::his5+-lox* | This study |
| UPY1284 | *W303A* + *bar1::LEU2, RAD52-YFP,*  *mcm2DENQ, ura3-1::URA3-pGal-mcm2DENQ (pUP989)* | This study |
| UPY1289 | *W303A* + *bar1::LEU2, RAD52-YFP +* pUP1230 *(P_GAL1_-*  *RNH1- URA3, 2 micron)* | This study |
| UPY1290 | *W303A* + *bar1::LEU2*, *RAD52-YFP*, *mcm2DENQ +* pUP1230 | This study |
| UPY1304 | *W303A* + *bar1::LEU2, RAD52-YFP*, *mrc1∆::his-lox,*  *∆pep4::KANMX* + pUP1230 | This study |
| UPY1336 | *W303A + bar1::hisG +pUP1230* | This study |
| UPY1337 | *W303A + bar1::hisG, mcm2DENQ + pUP1230* | This study |
| UPY1338 | *W303A + bar1::hisG, mrc1∆::loxP-his5^+^-loxP + pUP1230* | This study |
| UPY1339 | *W303A + bar1::hisG + pUP1242* | This study |
| UPY1340 | *W303A + bar1::hisG, mcm2DENQ + pUP1242* | This study |
| UPY1341 | *W303A + bar1::hisG, mrc1∆::loxP-his5^+^-loxP + pUP1242* | This study |
| UPY1342 | *W303A + bar1::LEU2, RAD52-YFP + pUP1242* | This study |
| UPY1343 | *W303A + bar1::LEU, RAD52-YFP, mcm2DENQ + pUP1242* | This study |
| UPY1344 | *W303A + bar1:LEU2, RAD52-YFP, mrc1∆::loxP-his5^+^-loxP + pUP1242* | This study |

**Table B** – Plasmids list

| Plasmid | Genes involved | Source |  |
| --- | --- | --- | --- |
| pUP989 | *P_GAL1_-mcm2DENQ, AMP, URA3,* | [3] |  |
| pUP1230 | *P_GAL1_-RNH1*, *AMP*, *URA3*, 2-micron | Open Biosystems [4] |  |
| pUP1242 | *P_GAL1_-TOP1*, *Amp*, *URA3*, 2-micron | Open Biosystems [4] | |

References

1. O'Connell K, Jinks-Robertson S, Petes TD. Elevated Genome-Wide Instability in Yeast Mutants Lacking RNase H Activity. Genetics. 2015;201(3):963-75. doi: 10.1534/genetics.115.182725. PubMed PMID: 26400613; PubMed Central PMCID: PMC4649664.

2. Chen C, Kolodner RD. Gross chromosomal rearrangements in Saccharomyces cerevisiae replication and recombination defective mutants. Nat Genet. 1999;23(1):81-5.

3. Tsai FL, Vijayraghavan S, Prinz J, MacAlpine HK, MacAlpine DM, Schwacha A. Mcm2-7 Is an Active Player in the DNA Replication Checkpoint Signaling Cascade via Proposed Modulation of Its DNA Gate. Mol Cell Biol. 2015;35(12):2131-43. doi: 10.1128/MCB.01357-14. PubMed PMID: 25870112; PubMed Central PMCID: PMC4438241.

4. Gelperin DM, White MA, Wilkinson ML, Kon Y, Kung LA, Wise KJ, et al. Biochemical and genetic analysis of the yeast proteome with a movable ORF collection. Genes Dev. 2005;19(23):2816-26. doi: 10.1101/gad.1362105. PubMed PMID: 16322557; PubMed Central PMCID: PMC1315389.
